# Participating in extracurricular activities and school sports during the COVID-19 pandemic: Associations with child and youth mental health

**DOI:** 10.3389/fspor.2022.936041

**Published:** 2022-08-29

**Authors:** Kaitlyn LaForge-MacKenzie, Katherine Tombeau Cost, Kimberley C. Tsujimoto, Jennifer Crosbie, Alice Charach, Evdokia Anagnostou, Catherine S. Birken, Suneeta Monga, Elizabeth Kelley, Christie L. Burton, Robert Nicolson, Stelios Georgiades, Daphne J. Korczak

**Affiliations:** ^1^Department of Psychiatry, Neuroscience and Mental Health, Hospital for Sick Children, Toronto, ON, Canada; ^2^Department of Psychiatry, Faculty of Medicine, University of Toronto, Toronto, ON, Canada; ^3^Department of Pediatrics, Faculty of Medicine, University of Toronto, Toronto, ON, Canada; ^4^Holland Bloorview Research Institute, Toronto, ON, Canada; ^5^Division of Paediatric Medicine, Hospital for Sick Children, Toronto, ON, Canada; ^6^Department of Psychiatry, Queens University, Kingston, ON, Canada; ^7^Department of Psychology, Faculty of Arts and Science, Queens University, Kingston, ON, Canada; ^8^Department of Psychiatry, Schulich School of Medicine and Dentistry, Western University, London, ON, Canada; ^9^Department of Psychiatry and Behavioural Neurosciences, Faculty of Health Sciences, McMaster University, Hamilton, ON, Canada

**Keywords:** extracurricular activities, school sports, children and youth, mental health, COVID-19 pandemic

## Abstract

In Ontario, Canada, school extracurricular activities and sports were modified or canceled for a prolonged period due to public health restrictions resulting from the COVID-19 pandemic. The present study aims to examine the association of changes to extracurricular and sport participation and child and youth mental health. Data were collected on child and youth mental health symptoms (*n* = 908) and participation in extracurricular activities and sports in the 2019–2020 and 2020–2021 academic years. Results indicated that pre-COVID (2019–2020) participation in either extracurricular activities or sports was associated with reduced anxiety, inattention, and hyperactivity during the pandemic (β range −0.08 to −0.11, *p* < 0.05). Participation in either extracurricular activities or sports during-COVID (2020–2021) was associated with lower depressive symptoms (β range −0.09 to −0.10, *p* < 0.05). Findings suggest that participation in extracurricular activities and/or school sports both before or during the COVID-19 pandemic were associated with better mental health outcomes in children and youth. Implications of this work consider future situations where restrictions on extracurricular and sport participation are reinstated and the impact of child and youth mental health.

## Introduction

Schools are places where children and youth are encouraged to learn, explore, and grow both socially and as individuals. Extracurricular activities (ECAs) and school sports offer experiential learning opportunities and help students to learn new skills, develop interests, and cultivate friendships. As part of their educational mandate, schools typically offer ECAs and sports. In the province of Ontario prior to the COVID-19 pandemic, 93.9% of elementary schools and 97.0% of secondary schools offered ECAs; 93.5% of elementary and 98.0% of secondary schools offered school sports (People for Education, 2021). However, as a result of the pandemic, ECAs and sports were either fully or partially removed from Ontario schools as part of public health safety directives (Borrelli, 2021; Fox, 2021; McKenzie-Sutter, 2021; People for Education, 2021). During the 2020-2021 academic year, 18.0% of elementary schools and 70.0% of secondary schools offered ECAs; only 5.0% of elementary and 8.0% of secondary schools offered school sports. By reducing or removing ECAs and sports from schools, the potential to benefit from these activities may have been subsequently impacted, including opportunities related to health, wellness, and development.

Participation in ECAs and sports have a positive impact on child and youth mental health and development (Eccles et al., 2003; Gilman et al., 2004; Jewett et al., 2014; Heath et al., 2018; Guilmette et al., 2019; Oberle et al., 2019a,b). Pre-COVID literature shows that students who participate in ECAs and sports experience lower levels of anxiety, depressive symptoms, and perceived stress (Jewett et al., 2014; Oberle et al., 2020) as well as higher levels of psychosocial well-being, satisfaction with life, self-esteem, self-image, and self-confidence compared with non-participating students (Harrison and Narayan, 2003; Gilman et al., 2004; Rodriguez-Ayllon et al., 2019; O'Donnell et al., 2020). Subsequently, when non-participating students become involved in ECAs and sports, they show better mental health outcomes over time facilitated by high peer belongingness; this highlights the importance of the associations between the social supports and connections afforded by ECAs and sports and improved child and youth mental health outcomes (Oberle et al., 2019a).

Considering ECAs specifically, children and youth who participate in ECAs gain positive psychosocial and emotional experiences that they can then carry into adulthood (Jewett et al., 2014; Guilmette et al., 2019). ECA participants have lower rates of substance-use compared with their peers (Harrison and Narayan, 2003) and have lower recreational screen-use after school compared to non-participants (Oberle et al., 2020). Further, different ECA activities are differentially associated with mental health. For example, children and youth who participated in arts or performance activities (i.e., music, drama, or visual arts) reported high levels of self-confidence, self-esteem, and belonging (Zarobe and Bungay, 2017) and low instances of risky behaviors such as alcohol-use (Eccles et al., 2003) while students who participated in academic clubs (i.e., debate team, chess, or tutoring) demonstrated high educational and occupational outcomes in early adulthood (Eccles et al., 2003).

With regard to school sport participation, student athletes showed lower levels of depression and psychological distress compared with their peers, and higher rates of body satisfaction, self-image, confidence, and physical, social, and emotional well-being (Harrison and Narayan, 2003; Jewett et al., 2014; Rodriguez-Ayllon et al., 2019; O'Donnell et al., 2020). The benefits of school sport participation are evident even in younger children, as elementary school students who participated in team (school) sports reported higher rates of emotional well-being compared with non-participants (Oberle et al., 2019b). In addition to the psychosocial and emotional benefits, school sports also promote physical activity and social development. Research confirmed the association of decreased physical activity and increased sedentary behavior with higher rates of depression in cross-sectional and longitudinal studies of children and adolescents (Korczak et al., 2017; Rodriguez-Ayllon et al., 2019). In addition to supporting options for physical activity, school sports provide opportunities to develop social skills and create a sense of community and belonging (O'Donnell et al., 2020). Student athletes demonstrated greater school attachment (Eccles et al., 2003), indicating that athletes were more likely to perceive supportive, meaningful relationships at school, which positively influenced their mental well-being (McLaughlin and Clarke, 2010). Student athletes also perform better academically, with sport positively impacting both memory and achievement (Lindner, 1999; Taras, 2005). Along with many of the shared benefits with ECAs, participation in school sports provide opportunities to engage in physical activity, improve social skills, and strengthen academic performance.

A key feature of schools-based ECAs and sports is that they are both accessible and affordable. Student participation in ECAs and sports is higher at schools that promoted activities regardless of ability (i.e., intramural programs), particularly for children from low-income and racialized families who may be at increased risk for negative health outcomes (Kanters et al., 2013). A recent review study on economically at-risk youth in ECA and sports programs showed that as participation rates increased for low-income students, educational, social, psychological, and behavioral outcomes improved. Increased participation resulted in greater academic (educational) and non-academic (psychosocial) outcomes in a dose-dependent fashion (Heath et al., 2018). Providing ECAs and sports in schools creates opportunities for students of all abilities and income-levels to participate, with associated improvements in mental health and academic achievement.

To our knowledge only one study has examined the impact of COVID-19 restrictions on student *sport* participation and mental health. Student athletes who were unable to participate in sports during the initial phases of the COVID-19 pandemic experienced higher levels of depression when compared to the pre-pandemic period (McGuine et al., 2020). However, this study only included adolescents (ages 13–19 years) and did not examine the impact of loss of *ECAs* on mental health. As such, the impact of COVID-19 restrictions on ECA/sport participation and mental health of students in elementary school and students participating in non-sport ECAs is currently undetermined.

## Hypothesis

The objective of the present study was to examine the associations of participating in ECAs and sports before (2019–2020) and during (2020–2021) the COVID-19 pandemic on children's mental health (MH) outcomes (depression, anxiety, hyperactivity, and inattention) during the pandemic. We explored the relationships of participating in ECAs/sports prior to and during the pandemic separately and hypothesized that participants would show fewer symptoms of depression, anxiety, hyperactivity and inattention than non-participants.

## Methods

### Participants

Data for these analyses were collected as part of the Ontario COVID-19 and Kids Mental Health study, a collaboration of four established research cohorts with pre-existing participant bases. Two cohorts are recruited though clinically-referred pathways (i.e., mental health and neurodevelopmental diagnoses) and two are recruited within the broader community (Korczak et al., 2022). Children and adolescents from the SickKids Psychiatry cohort are referred to an outpatient clinic for MH evaluations of disorders including but not limited to depression, anxiety, attention deficit/hyperactivity disorder (ADHD), obsessive-compulsive disorder (OCD), and disruptive behaviors (Korczak et al., 2022). Children and youth in the Province of Ontario Neurodevelopmental Disorder (POND) cohort have diagnoses of neurodevelopmental disorders (NDDs), including autism spectrum disorders (ASD), ADHD, OCD, and intellectual disabilities (About POND, 2021). Children in The Applied Research Group for Kids (TARGet Kids!) cohort are recruited from primary care practices in the Greater Toronto Area from birth to 5 years of age, and followed at their primary care visits (Carsley et al., 2015). Children and adolescents in the population-based Spit for Science cohort are recruited at an urban science museum (Spit for Science, 2021). Information including detailed descriptions of the cohorts, consent, and participation processes are described in Korczak et al. (2022).

### Procedure

Questionnaires were completed by parents and children (ages 10–18 years) at two timepoints by online survey using REDCap (Research Electronic Data Capture; Harris et al., 2009, 2019). Parents reported on their children ages 6 years (Grade 1) and older (i.e., school-aged) (Korczak et al., 2022). Data on ECA and sport participation were collected in November 2020 by parent-report on child participation in ECAs/sports during in-person learning in Fall 2020 and child participation in ECAs/sports the previous academic year (2019–2020). Data on current ADHD symptoms (i.e., the previous 2 weeks) were collected in November 2020; current depression and anxiety symptoms (i.e., the past 2 weeks) were collected in February and March, 2021. The study was approved by the institutional ethics boards at all participating sites (REB # 100070222). All participants provided informed consent.

### Measures

#### ECA and sport participation measures

Parents reported on the frequency with which their child participated in *sports or teams* or *clubs or activities* during the 2019–2020 and the 2020–2021 school year using a 5-point ordinal scale (1 = most days, 2 = a few times a week, 3 = once a week, 4 = about once a month, 5 = almost never, and not applicable [N/A]). Responses were grouped, considering limitations of ordinal scales (i.e., unequal interval measurement), such that responses of 1, 2, or 3 indicated regular participation in ECAs and sports and responses of 4, 5, and N/A indicated that their child rarely or did not participate in ECAs and sports.

#### Mental health measures

***Revised Children's Anxiety and Depression Scale – Parent-Report*** (RCADS-P; Ebesutani et al., 2015). Depression was assessed using the 10-item major depressive disorder (MDD) subscale of the RCADS, rating frequency of symptoms on a 4-point scale (1 = never; 4 = always). Higher scores indicate higher levels of depressive symptoms, with borderline clinical *t*-scores ranging from 65 and 69 and clinically significant *t*-scores of 70 and above.

***Screen for Child Anxiety Related Disorders*** (SCARED; Birmaher et al., 1997). Anxiety was assessed using the 9-item generalized anxiety disorder (GAD) subscale of the SCARED, rating items on 3-point scale (0 = not true or hardly true; 2 = very true or often true). Higher scores indicate higher levels of anxiety symptoms, with the clinical threshold for GAD subscale being a score of 9 out of a possible 18.

***Strengths and Weaknesses of Attention-Deficit/Hyperactivity Disorder Symptoms and Normal Behavior Scale*** (SWAN; Swanson et al., 2012). Inattention and hyperactivity were assessed the 18 item SWAN instrument. The measure employs a 7-point rating scale (−3 = far below average; 3 = far above average) in which parents compare their child to other children of the same age for each item. In the original scoring, higher scores indicate lower symptoms of ADHD and as such, coding was reversed such that a higher score indicted higher levels of inattentive and hyperactivity symptoms.

### Confound measures

Demographic data included both family (parent) and child information such as household annual income, child ethnicity, and child age.

### Statistical analyses

In order to ensure independence of observations, one child per family was included in the analyses. Where more than one child per family participated in the study, one child was chosen based on the following algorithm: older child age, more complete data, greater ECA/sport participation (if eldest siblings were the same age), and finally, random selection (if all other conditions were equal, one sibling was chosen at random).

Four hierarchical linear regression models assessed the impact of ECA and sport participation on each mental health outcome (1) depression, (2) anxiety, (3) inattention, and (4) hyperactivity. Child age, child ethnicity, and household income were entered in the first block; pre-COVID activity participation in block two; and during-COVID activity participation in block three. Multiple imputation using logistic regression (*n* = 15) at the item-level was used for missing predictor and covariate data where missingness was approximately 15% (Bodner, 2008; White et al., 2011). Analyses with complete cases (listwise deletion) are available in the Supplement. Statistical analyses were conducted using both R (Team, 2015, 2018) and SPSS 27 (IBM Corp, 2020).

## Results

A total of 908 children and youth were included in this study (mean age = 10.77 years [*SD* = 3.77]; 496 [55%] male). Nearly two-thirds of participants were of European-North American (*n* = 574; 65%) descent. Non-European-North American (e.g., Indigenous, Black, Latin, Caribbean, Asian, or Other; *n* = 155 or 18%) and multiple ethnic backgrounds (*n* = 155 or 18%) comprised the remainder of children's ethnicity. See Table 1 for a more detailed description of the participant characteristics and mean MH outcomes.

**Table 1:** Sample characteristics and demographics and mean mental health scores of children and youth by parent-reported analyses.

**Variables**	**Categories**	**Total sample**	**Elementary school**	**High school**
		***n*** **= 908**	***n*** **= 662**	***n*** **= 246**
Age (years)		*M* = 10.77	*M* = 9.1	*M* = 15.08
		(*SD* = 3.35)	(*SD* = 2.06)	(*SD* = 1.34)
Sex at birth	Male	496 (55%)	374	108
	Female	410 (45%)	287	117
	Missing	2 (0%)	1	1
Ethnicity	European/North American	574 (64%)	408	153
	Non-European/North American	155 (18%)	125	27
	Multiple	155 (18%)	112	40
	Missing	24 (3%)	17	7
Household income	>$80,000	553 (71%)	402	135
	< $80,000	224 (29%)	164	56
Pre-COVID MH/NDD diagnosis	MH/NDD Diagnoses	554 (61%)	353	201
	No diagnoses	354 (39%)	309	45
Pre-COVID extracurricular participation	Yes	513 (58%)	380	133
	No	366 (42%)	258	108
	Missing	29 (3%)	24	3
During-COVID extracurricular participation	Yes	99 (16%)	65	34
	No	530 (84%)	389	141
	Missing	279 (31%)	208	71
Pre-COVID sport participation	Yes	511 (56%)	404	107
	No	369 (41%)	235	134
	Missing	28 (3%)	23	5
During-COVID sport participation	Yes	167 (27%)	153	14
	No	450 (73%)	317	133
	Missing	291 (32%)	192	99
Depression (RCADS-P *t*-score)		*M* = 62.29 (*SD* = 18.42)	*M* = 61.68 (*SD* = 18.66)	*M* = 64.30 (*SD* = 17.78)
Anxiety (SCARED score)		*M* = 7.38 (*SD* = 5.35)	*M* = 6.85 (SD = 5.22)	*M* = 8.89 (SD = 5.38)
Hyperactivity (SWAN subscale)		*M* =0.10 (*SD* = 11.73)	*M* =0.93 (*SD* = 11.72)	*M* = −2.10 (*SD* = 11.61)
Inattention (SWAN subscale)		*M* = 2.90 (*SD* = 12.33)	*M* = 2.79 (*SD* = 12.12)	*M* = 3.60 (*SD* = 12.92)

Household income represents annual income in Canadian dollars; MH, mental health; NDD, neurodevelopmental disorder; RCADS-P, Revised Child and Anxiety Depression Scale-Parent Version; SCARED, Screen for Child Anxiety Related Disorders; SWAN, Strengths and Weaknesses of Attention-Deficit/Hyperactivity Disorder Symptoms and Normal Behavior Scale.

During the 2019–2020 academic year (hereafter referred to as “pre-COVID”), the majority of children and youth participated in ECAs, 58% (*n* = 513) and sports, 58% (*n* = 511) at least 1 day per week. In November 2020 (herein labeled “during-COVID”), participation rates decreased to 16% (*n* = 99) for ECAs and 27% (*n* = 167) for sports. During-COVID ECA participants were more often females (57% vs 48% pre-COVID), from households with annual income < $80,000 (68%), and of non-European/North-American descent (27% vs. 20% pre-COVID). During-COVID sport participants were more often non-European/North-Americans (26% vs. 21% pre-COVID). Mean MH measures for each participation group are found in Table 2. Chi-square tables showing differences in group composition are found in Supplementary Table 1 full details regarding pre-COVID and during-COVID ECA and sport participant and non-participant characteristics are found in Supplementary Table 2.

**Table 2:** Mean current mental health scores by domain and measure in pre-COVID and during-COVID extracurricular activity and sport participants and non-participants.

**Pre-COVID participant and non-participant mental health outcomes** ^*^				
	**Pre-COVID** **ECA participants** **(*n* = 513)**	**Pre-COVID** **ECA non-participants** **(*n* = 366)**	**Pre-COVID** **sport participants** **(*n* = 511)**	**Pre-COVID** **sport non-participants** **(*n* = 369)**
Depression (RCADS-P *t*-score)	*M* = 61.44 (*SD* = 18.96)	*M* = 63.31 (*SD* = 17.60)	*M* = 61.02 (*SD* = 19.18)	*M* = 64.13 (*SD* = 17.38)
Anxiety (SCARED score)	*M* = 7.02 (*SD* = 5.37)	*M* = 7.89 (*SD* = 5.32)	*M* = 6.85 (*SD* = 5.26)	*M* = 8.12 (*SD* = 5.43)
Hyperactivity (SWAN subscale)	*M* = −1.03 (*SD* = 11.61)	*M* = 1.56 (*SD* = 11.64)	*M* = −0.26 (*SD* = 11.63)	*M* = 0.40 (*SD* = 11.59)
Inattention (SWAN subscale)	*M* =0.95 (*SD* = 12.39)	*M* = 5.49 (*SD* = 11.82)	*M* = 1.62 (*SD* = 12.47)	*M* = 4.48 (*SD* = 11.73)
**During-COVID Participant and Non-Participant Mental Health Outcomes** ^*^				
	**During-COVID** **ECA participants** **(*****n*** = **99)**	**During-COVID** **ECA non-participants** **(*****n*** = **530)**	**During-COVID** **sport participants** **(*****n*** = **167)**	**During-COVID** **sport non-participants** **(*****n*** = **450)**
Depression (RCADS-P t-score)	*M* = 55.90 (*SD* = 18.65)	*M* = 62.81 (*SD* = 17.72)	*M* = 59.22 (*SD* = 19.27)	*M* = 63.66 (*SD* = 18.48)
Anxiety (SCARED score)	*M* = 6.98 (*SD* = 5.82)	*M* = 7.43 (*SD* = 5.35)	*M* = 5.94 (*SD* = 4.78)	*M* = 7.74 (*SD* = 5.52)
Hyperactivity (SWAN subscale)	*M* = −2.40 (*SD* = 12.32)	*M* = 0.53 (*SD* = 11.36)	*M* = 0.07 (*SD* = 11.06)	*M* = 0.70 (*SD* = 11.50)
Inattention (SWAN subscale)	*M* = −0.88 (*SD* = 12.34)	*M* = 3.49 (*SD* = 12.15)	*M* = 1.11 (*SD* = 11.17)	*M* = 3.96 (*SD* = 12.50)

* All current mental health scores were collected from November 2020 to February 2021; RCADS-P, Revised Child and Anxiety Depression Scale-Parent Version; SCARED, Screen for Child Anxiety Related Disorders; SWAN, Strengths and Weaknesses of Attention-Deficit/Behavior Scale Hyperactivity Disorder Symptoms and Normal.

### Depression

After controlling for confounders, pre-COVID ECA participation was not significantly related to levels of depression during the pandemic. However, after controlling for confounders, during-COVID ECA participation (β = −0.09, 95% CI [-10.22, −1.39], *p* = 0.01,) was significantly associated with decreased levels of depression in February 2021 and added significant variance to Block 3 as detailed in Table 3.

**Table 3:** The associations of activity participation and MH outcomes using multiple imputation hierarchical linear regression analyses.

		**Depression**						**Anxiety**						**Hyperactivity**						**Inattention**					
**EA**	**Variable**	**β**	**SE**	**LCI, UCI**	***p***	***R^2^***	**Δ*R^2^***	**β**	**SE**	**LCI, UCI**	***p***	***R^2^***	**Δ*R^2^***	**β**	**SE**	**LCI, UCI**	***p***	***R^2^***	**Δ*R^2^***	**β**	**SE**	**LCI, UCI**	***p***	***R^2^***	**Δ*R^2^***
	***Block 1***					0.01	–					0.02	**–**					0.01	**–**					0.01	–
	Intercept	**–**	**2.82**	**53.00**, **64.05**	<0.001*			**–**	**0.15**	**−0.67**, **−0.09**	0.01*			**–**	**1.50**	**2.12**, **8.00**	<0.001*			**–**	1.57	−1.04, 5.13	0.19		
	Age	**0.09**	**0.22**	**0.10**, **0.95**	0.02*			**0.15**	**0.01**	**0.02**, **0.07**	<0.001*			**−0.08**	**0.12**	**−0.50**, **−0.05**	0.02*			0.06	0.12	−0.01, 0.47	0.07		
	Income	−0.06	1.73	−5.97, 0.73	0.13			−0.04	0.09	−0.27, 0.07	0.26			**−0.08**	**0.91**	**−4.19**, **−0.64**	0.01*			−0.05	0.93	−3.38, 0.28	0.10		
	Ethnicity	−0.01	0.82	−1.77, 1.46	0.85			−0.07	0.04	−0.17, 0.01	0.07			−0.04	0.46	−1.42, 0.36	0.25			−0.06	0.48	−1.74, 0.14	0.09		
	***Block 2***					0.01	0.00					0.02	0.00					**0.02**	0.01*					**0.04**	0.03*
	Intercept	**–**	**2.92**	**53.69**, **64.05**	<0.001*			**–**	**0.15**	**−0.60**, **0.00**	<0.05*			**–**	**1.55**	**3.32**, **9.40**	<0.001*			**–**	**1.61**	**1.17**, **7.50**	0.007*		
	Age	**0.09**	**0.22**	**0.10**, **0.95**	0.02*			**0.14**	**0.01**	**0.02**, **0.07**	<0.001*			**−0.08**	**0.12**	**−0.51**, **−0.06**	0.02*			0.06	0.12	−0.02, 0.45	0.08		
	Income	−0.06	1.71	−5.82, 0.90	0.15			−0.03	0.09	−0.26, 0.09	0.33			**−0.07**	**0.91**	**−3.98**, **−0.43**	0.02*			−0.05	0.92	−2.97, 0.63	0.20		
	Ethnicity	0.00	0.83	−1.72, 1.52	0.91			−0.06	0.04	−0.16, 0.01	0.09			−0.04	0.46	−1.35, 0.44	0.32			−0.05	0.47	−1.60, 0.26	0.16		
	Pre-COVID participation	−0.04	1.40	−4.35, 1.14	0.25			−0.06	0.08	−0.29, 0.01	0.07			**−0.10**	**0.80**	**−3.98**, **−0.84**	0.003*			**−0.09**	**0.83**	**−5.91**, **−2.65**	<0.001*		
	***Block 3***					**0.02**	0.01*					0.02	0.01					0.02	0.00					0.04	0.00
	Intercept	**–**	**2.93**	**53.70**, **65.17**	<0.001*			**–**	0.15	−0.60, 0.00	0.05			**–**	**1.55**	**3.33**, **9.45**	<0.001*			**–**	1.62	1.20, 7.53	0.007		
	Age	**0.10**	**0.22**	**0.12**, **0.97**	0.01*			**0.15**	**0.01**	**0.02**, **0.07**	<0.001*			**−0.08**	**0.12**	**−0.50**, **−0.05**	0.02*			0.02	0.12	−0.02, 0.46	0.07		
	Income	−0.06	1.73	−6.07, 0.76	0.13			−0.03	0.09	−0.26, 0.08	0.31			**−0.08**	**0.91**	**−4.05**, **−0.49**	0.01*			−0.04	0.93	−3.07, 0.56	0.18		
	Ethnicity	0.00	0.82	−1.62, 1.60	0.99			−0.06	0.04	−0.16, 0.01	0.09			−0.04	0.46	−1.33, 0.46	0.34			−0.05	0.47	−1.57, 0.28	0.17		
	Pre-COVID participation	−0.01	1.46	−3.30, 2.43	0.79			−0.06	0.08	−0.28, 0.03	0.12			**−0.08**	**0.84**	**−3.66**, **−0.37**	0.02*			**−0.08**	**0.87**	**−5.49**, **−2.10**	<0.001*		
	During-COVID participation	**−0.09**	**2.24**	**−10.22**, **−1.39**	0.01*			−0.03	0.12	−0.30, 0.15	0.51			−0.06	1.25	−4.52, 0.60	0.13			−0.07	1.36	−5.06 , 0.28	0.08		
**Sports**	**Variable**	β	**SE**	**LCI, UCI**	***p***	***R** ^2^*	Δ***R**^2^*	β	**SE**	**LCI, UCI**	***p***	***R** ^2^*	**Δ** ***R** ^**2**^*	***β***	**SE**	**LCI, UCI**	***p***	***R** ^2^*	Δ***R**^2^*	β	**SE**	**LCI, UCI**	***p***	***R** ^2^*	**Δ** ***R** ^**2**^*
	***Block 1***					0.01	**–**					0.02	**–**					0.01	**–**					0.01	**–**
	Intercept	**–**	**2.80**	**53.20**, **64.18**	<0.001*			**–**	**0.15**	**−0.65**, **−0.08**	0.01*			**–**	**1.49**	**2.18**, **8.02**	<0.001*			**–**	1.58	−1.02, 2.17	0.19		
	Age	**0.09**	**0.22**	**0.10**, **0.95**	0.02*			**0.15**	**0.01**	**0.02**, **0.07**	<0.001*			**−0.08**	**0.12**	**−0.51**, **−0.05**	0.02*			0.06	0.12	−0.02, 0.46	0.07		
	Income	−0.06	1.64	−6.02, 0.42	0.09			−0.05	0.09	−0.29, 0.05	0.17			**−0.09**	**0.88**	**−4.08**, **−0.63**	0.01*			−0.06	0.93	−3.36, 0.30	0.10		
	Ethnicity	−0.01	0.83	−1.79, 1.44	0.83			−0.07	0.04	−0.17, 0.00	0.06			−0.05	0.46	−1.50, 0.29	0.18			−0.06	0.48	−1.80, 0.09	0.08		
	***Block 2***					0.01	0.00					**0.04**	0.02*					0.01	0.00					**0.02**	0.01*
	Intercept	**–**	**2.98**	**54.72**, **66.39**	<0.001*			**–**	0.16	−0.48, 0.13	0.26			**–**	**1.60**	**2.48**, **8.75**	<0.001*			**–**	**1.69**	**0.57**, **7.18**	0.02*		
	Age	**0.08**	**0.22**	**0.05**, **0.91**	0.03*			**0.13**	**0.01**	**0.02**, **0.06**	<0.001*			**−0.08**	**0.12**	**−0.52**, **−0.06**	0.01*			0.05	0.12	−0.07, 0.41	0.16		
	Income	−0.06	1.65	−5.91, 0.57	0.11			−0.05	0.09	−0.27, 0.07	0.23			**−0.10**	**0.88**	**−4.08**, **−0.63**	0.01*			−0.05	0.93	−3.22, 0.41	0.13		
	Ethnicity	0.00	0.83	−1.65, 1.60	0.98			−0.05	0.04	−0.15, 0.02	0.12			−0.04	0.46	−1.47, 0.33	0.21			−0.05	0.48	−1.68, 0.21	0.13		
	Pre-COVID participation	−0.07	1.41	−5.34, 0.19	0.07			**−0.12**	**0.08**	**−0.40**, **−0.10**	<0.001*			−0.03	0.81	−2.29, 0.87	0.38			**−0.10**	**0.85**	**−4.14**, **−0.81**	0.004*		
	***Block 3***					**0.02**	0.01*					0.04	0.00					0.01	0.00					0.02	0.00
	Intercept	**–**	**2.99**	**55.44**, **67.16**	<0.001*			**–**	0.16	−0.46, 0.16	0.36			**–**	**1.62**	**2.62**, **8.95**	<0.001*			**–**	1.70	0.78, 7.46	0.02		
	Age	0.07	0.22	−0.01, 0.85	0.06			**0.13**	**0.01**	**0.02**, **0.06**	<0.001*			**−0.09**	**0.12**	**−0.54**, **0.07**	0.01*			0.04	0.12	−0.09, 0.40	0.22		
	Income	−0.07	1.64	−5.94, 0.48	0.10			−0.04	0.09	−0.28, 0.06	0.22			**−0.10**	**0.88**	**−4.10**, **−0.65**	0.01*			−0.05	0.93	−3.26, 0.38	0.12		
	Ethnicity	0.00	0.83	−1.59, 1.66	0.97			−0.05	0.04	−0.15, 0.02	0.13			−0.04	0.46	−1.46, 0.34	0.23			−0.05	0.48	−1.66, 0.24	0.14		
	Pre-COVID participation	−0.03	0.1.58	−4.26, 1.92	0.46			**−0.11**	**0.09**	**−0.38**, **−0.04**	0.02*			−0.02	0.90	−2.18, 1.33	0.64			**−0.08**	**0.95**	**−3.92**, **−0.18**	0.03*		
	During-COVID participation	**−0.10**	**1.88**	**−7.50**, **−0.11**	0.04*			−0.05	0.11	−0.32, 0.09	0.28			−0.03	1.08	−2.91, 1.33	0.47			−0.03	1.18	−3.47, 1.17	0.33		

**Block 2** controlled for age, income, ethnicity, with pre-COVID participation as the independent variable; **Block 3** controlled for age, income, ethnicity, pre-COVID participation with during-COVID participation as the independent variable; Age = child age in years; Income = annual household income in Canadian dollars; Depression was measured by the Revised Child and Anxiety Depression Scale-Parent Version (RCADS-P); Anxiety was measured by the Screen for Child Anxiety Related Disorders (SCARED); Hyperactivity and inattention were measured by subscales of the Strengths and Weaknesses of Attention-Deficit/Hyperactivity Disorder Symptoms and Normal Behavior Scale. Bold values indicate statistical significance p < 0.05. Asterisk indicates statistical significance p < 0.05.

**Figure 1:**
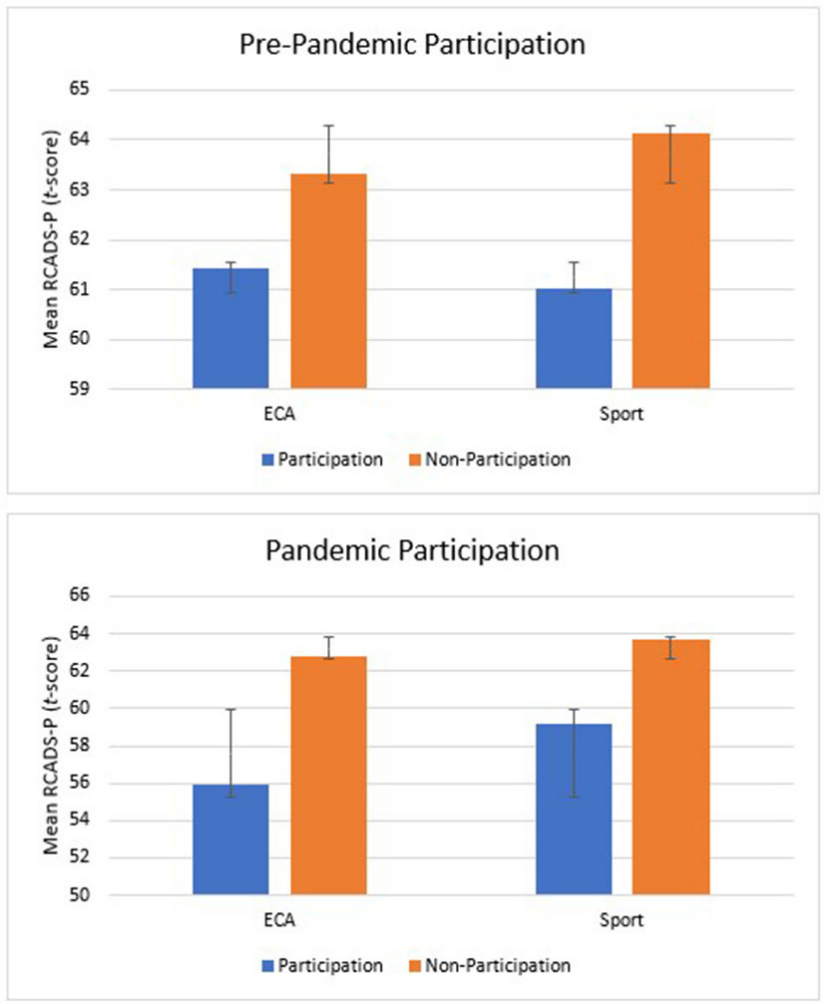
Mean depressive symptoms in extracurricular activities and sports pre-COVID and during-COVID participation. Depressive symptoms were reported by parents though the RCADS-P (Revised Child and Anxiety Depression Scale-Parent Version); Error bars represent standard error.

Similar to ECAs, after controlling for confounders, pre-COVID sport participation was marginally but ultimately non-significantly associated with levels of depression in February 2021 in Block 2 (*p* =0.07; see Table 3). During-COVID sport participation was inversely associated with child and youth depression (β = −0.10, 95% CI [−7.50, −0.11], *p* < 0.05) and added significant variance to Block 3. Together these findings suggest that children and youth who participated in ECAs and/or sports in November 2020 had lower levels of depressive symptoms in February 2021 than those students who did not participate in ECAs and/or sports (Figure 1; Table 3).

### Anxiety

With respect to ECA participation, neither the pre-COVID ECA model nor during-COVID ECA model were significant.

**Figure 2:**
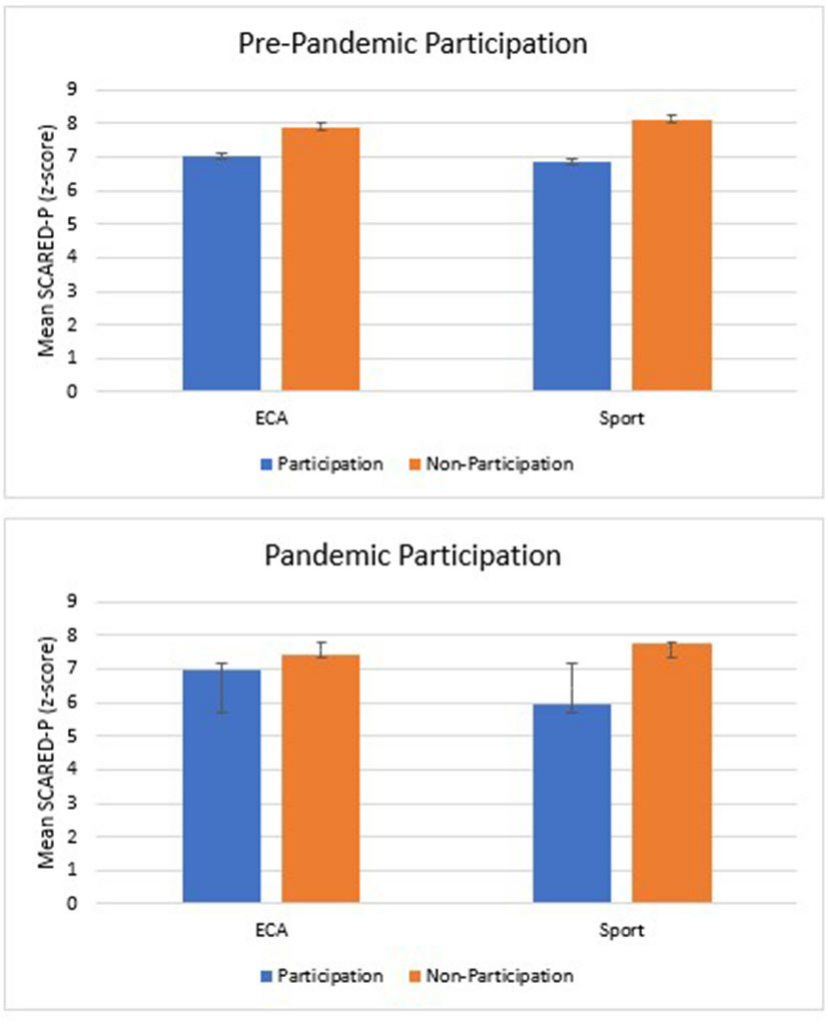
Mean anxiety symptoms in school sports comparing pre-COVID and during-COVID participation. Anxiety symptoms were reported by parents though the SCARED (Screen for Child Anxiety Related Disorders); Error bars represent standard error.

Pre-COVID sport participation was significantly associated with decreased levels of anxiety in February 2021 (β = −0.12, 95% CI [−0.40, −0.10], *p* < 0.001,) and explained significantly more variance than confounders alone. However, the model intercept was not significant and as such, these results along with those in Block 3, are not interpretable. These findings suggest that participation in either ECAs or sports in November 2020 was not associated with decreased anxiety in February 2021, although there may be a small association between lower levels of anxiety and pre-COVID sport participation (Figure 2; Table 3).

### Hyperactivity

**Figure 3:**
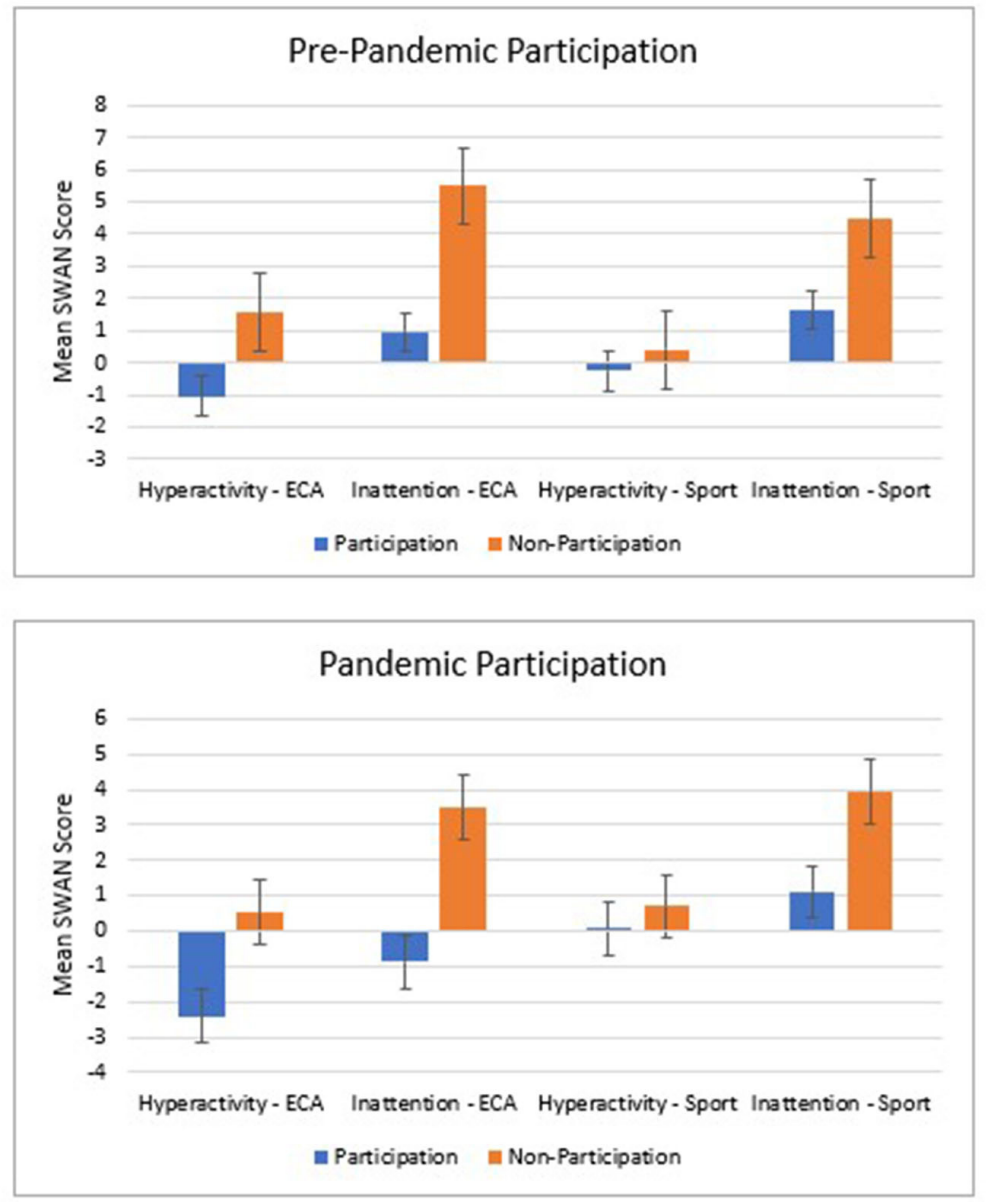
Mean hyperactivity and inattention symptoms in extracurricular activities and sports pre-COVID and during-COVID participation. ECA, extracurricular activities; Hyperactivity and inattention symptoms were reported by parents though the SWAN (Strengths and Weaknesses of ADHD symptoms) subscales; Error bars represent standard error.

After controlling for confounders, pre-COVID ECA participation was significantly associated with decreased levels of hyperactivity in November 2020 (β = −0.08, 95% CI [−3.66, −0.37], *p* = 0.02) and added significant variance in Block 2 (*p* = 0.003). During-COVID ECA participation did not have a significant association with levels of hyperactivity. This suggests that students who participated in ECAs prior to COVID-19 during the 2019–2020 academic year had lower reported levels of hyperactivity than students who did not participate in ECAs in the 2019–2020 academic year (Figure 3; Table 3).

After controlling for confounders, neither pre-COVID nor during-COVID sport participation were associated with levels of hyperactivity despite the models having statistical significance.

### Inattention

After controlling for confounders, pre-COVID ECA participation was significantly associated with decreased levels of inattention in November 2020 (β = −0.08, 95% CI [-5.41, −1.93], *p* < 0.001,) and added significant variance in Block 2 (*p* < 0.001). Although both the overall model (*p* =0.007 vs. Bonferroni correction *p* < 0.00625) and during-COVID ECA participation (*p* = 0.08) approached significance, neither reached statistical significance in Block 3.

After controlling for confounders, pre-COVID sport participation was significantly associated with decreased level of inattention in November 2020 (β = −0.08, 95% CI [−3.92, −0.18], *p* = 0.03) and added significantly more variance than confounders alone in Block 2 (*p* = 0.004). During-COVID sport participation was not significantly related with November 2020 levels of attention. In addition, the overall model (Block 3) was not significant (*p* = 0.02).

These results suggest that (despite non- or marginal-significance in the overall models) children and youth who participated in ECAs and/or sports in the school year prior to COVID-19 had fewer symptoms of inattention in November 2020 than children and youth who did not participate in ECAs and/or sports pre-COVID (Figure 3; Table 3).

## Discussion

The results of the present analysis indicate that participation in ECAs and school sports were associated with better child and youth MH during the first years of the COVID-19 pandemic. Students who participated in ECAs and school sports during the pandemic were reported to have lower depressive symptoms compared with non-participants. Students who participated in ECAs in the school year prior to the pandemic showed lower inattention and hyperactivity during pandemic school periods compared with pre-pandemic non-participants. Students who participated in school sports prior to the pandemic were reported to have lower inattentive symptoms and marginally lower anxiety compared with pre-pandemic non-participants. We find support for our hypothesis that children and youth who participated in ECAs and school sports, whether pre-COVID, during-COVID, or both, would have better mental health outcomes during the pandemic than students who were non-participants.

Participation in ECAs and sports were associated with decrease in MH symptoms during the pandemic. Pre-COVID ECA participation was associated with decreased hyperactivity and inattention symptoms and during-COVID ECA participation was associated with decreased depressive symptoms. Additionally, pre-COVID sport participation was associated with decreased inattention symptoms and to a certain extent, anxiety, while during-COVID sport participation was associated with decreased depression symptoms. These results suggest that ECA and sport participation has the potential to attenuate some of the negative consequences of the pandemic on child and youth MH. These findings align with previous research which showed that ECA and sports participants had fewer negative MH outcomes compared to their peers (Zarrett et al., 2009; Jewett et al., 2014; White et al., 2017; Rodriguez-Ayllon et al., 2019), and that these outcomes have the potential to carry forward into the future (Jewett et al., 2014; Guilmette et al., 2019). Furthermore, the sport participation findings support recent studies that show the benefit of middle childhood participation in school sports in lessening ADHD symptoms (Pagani et al., 2020).

The availability of ECAs and sports may be particularly important to mitigate the MH impacts as the COVID-19 pandemic continues, transitions, and recovers. As ECAs and sports canceled and/or significantly altered (i.e., operating virtually or employing “cohorting” as described in the Ontario Physical Activity Safety Standards in Education, 2021), this created additional strain on top of the already deteriorating MH of children and adolescents (Racine et al., 2021; Tombeau Cost et al., 2021) and stripped them of a key protective factor. A full restart of ECAs and sports in Ontario and regularizing their administration and operation (i.e., functioning in as much normal capacity as public health regulations allow) has potential to bolster and to prevent further degradation of child and youth mental health (Kanters et al., 2013; Heath et al., 2018). Cancellation or significant alteration of ECAs and sports as part of school safety mandates should be carefully evaluated in future situations, considering their removal on the state of MH in children and adolescents.

Participating in ECAs and school sports may be associated with better MH in several respects including decreased sedentary screen time, increased physical activity, and/or increased sense of community. ECA participants engaged in less recreational screen-use following school than non-participants (Oberle et al., 2020). This is relevant given the recent research during the pandemic suggested that child and youth screen-use increased and was detrimental to MH during the first years of the COVID-19 pandemic (Li et al., 2021). Disrupting school sports may also remove the means for affordable and accessible physical activity. Prior to the pandemic, less physical activity and more sedentary behaviors were linked to greater depression in children and youth (Korczak et al., 2017; Rodriguez-Ayllon et al., 2019; Guerrero et al., 2020). With opportunities afforded for physical activity to most students regardless of ability or income (Kanters et al., 2013; Heath et al., 2018), the suspension of school sports can reinforce the connection between sedentary behaviors and greater depressive symptoms.

In addition to positive health behaviors (i.e., less screen time and more physical activity), ECAs and sports also foster a sense of community, belonging, and connectedness to school, positively influencing MH (O'Donnell et al., 2020). At times where physical and social distancing were mandated and enforced, social community and connectedness may be especially important for child and youth MH (Tombeau Cost et al., 2021). Engaging in ECAs or playing school sports during pandemic periods may heighten a sense of belonging and create a protective environment at school against negative MH outcomes, particularly depression (Bauer et al., 2018; Oberle et al., 2019a; O'Donnell et al., 2020). A recent commentary support continued ECA and sport participation in schools to provide children and youth with socialization and connectedness which have been lacking during the pandemic (Lang, 2021). Future research can examine the mechanisms by which ECA and sport participation is associated with child and youth MH during and after the COVD-19 pandemic.

The present analysis has several strengths. The sample was drawn from both clinical and community populations for a diverse sample of children and youth and greater representation of MH in the broader population. This study is also unique in that it is a large scale, longitudinal study that includes a range of quantitative MH outcomes over the course of an international pandemic, which will allow the examination of these associations over time (Korczak et al., 2022).

The study also has several important limitations. Firstly, we did not measure the specific activities in which students were engaged and how they participated in these activities during the COVID-19 pandemic (i.e., ECAs vs. sports; in-person indoors vs.in-person outdoors vs.virtually vs. mixed). There is the potential that while ECAs and sports were offered in schools during COVID-19, participation did not have as much of a positive, MH impact due to the differences in activities and altered format of their operation. For example, participating in sports may offer different benefits than participating in ECAs. Further, participating virtually for training or working out would not have the same impact as participating with a team, in-person. This was not limited to sports. In-person meetings, band/choir practices, and performance arts also suffered if virtual participation was mandated or if they occurred at all. Children and youth continued to be physically and socially isolated from their peers. Secondly, we did not examine whether children and youth continued to participate in activities, lost activities, or never did activities; currently the groups were identified solely as “participation” and “no participation.” There may be differences in MH outcomes in the “no participation” group in terms of who lost vs. who never participated in ECAs and sports, specifically in how they perceive the lack of ECAs and sports in schools. Future research should examine which activities are running and/or are canceled, how children and youth are participating in these activities, and whether losing or never having ECAs and sports results in differences in MH outcome among these groups. Lastly, as the data in this study are cross-sectional, data regarding pre-COVID sport participation are subject to retrospective reporting bias. However, parents may be just as (or more) likely to “miss” a prior activity than to over-report involvement. As such, findings of the current study may underestimate the strength of the association between participation and MH outcomes.

## Conclusions

This study finds that participation in ECAs and sports are associated with better MH outcomes in children and youth. As permissions are once again afforded for ECAs and sports to operate in schools, Ontario school boards should recognize their importance to good MH in children and youth. Re-implementing and regularizing these activities in schools may offer an available, cost-effective option to support the MH for a large proportion of students. This work further points to policy implications for future situations where restriction on sports and ECAs are considered. These activities have the opportunity to foster a sense of normalcy with options to socialize, get physical activity, and reduce screen time—all of which may improve MH outcomes as the pandemic continues and transitions for Ontario children and youth.

## Data availability statement

The datasets presented in this article are not readily available because the dataset contains identifiable patient health information. Requests to access the datasets should be directed to DK, daphne.korczak@sickkids.ca.

## Ethics statement

The studies involving human participants were reviewed and approved by Research Ethics Board, Hospital for Sick Children. Written informed consent to participate in this study was provided by the participants' legal guardian/next of kin.

## Author contributions

KL-M conceived design of the analyses, supported design of data collection instruments, and drafted initial manuscript. KL-M, KTs, KTo, and DK designed, performed, and interpreted statistical analyses. EA, CB, AC, KTo, JC, SM, and DK initiated the larger project, designed and selected data collection measures, and created data collection procedures. EK, CB, RN, and SG supported design of larger project as well as data collection measures and procedures. EA, CB, AC, KTo, JC, SM, DK, KTs, and KL-M monitored data collection. EA, CB, AC, KTo, KTs, JC, SM, DK, EK, CB, RN, SG, and KL-M revised and approved final manuscript. All authors contributed to the article and approved the submitted version.

## Funding

The research of the Ontario COVID and Kids Mental Health Collaboration was funded by the Canadian Institutes for Health Research (#173092); the Ontario Ministry of Health (#700); Centre of Brain and Mental Health, SickKids; Leong Centre for Healthy Children, SickKids; and the Miner's Lamp Innovation Fund in Prevention and Early Detection of Severe Mental Illness, University of Toronto. In-kind support was provided by the Ontario Brain Institute for all POND data.

## Conflict of interest

The authors declare that the research was conducted in the absence of any commercial or financial relationships that could be construed as a potential conflict of interest.

## Publisher's note

All claims expressed in this article are solely those of the authors and do not necessarily represent those of their affiliated organizations, or those of the publisher, the editors and the reviewers. Any product that may be evaluated in this article, or claim that may be made by its manufacturer, is not guaranteed or endorsed by the publisher.

## Author disclaimer

The views of the Ontario COVID and Kids Mental Health Collaboration do not necessarily represent those of the Province on Ontario and the Ontario Ministry of Health.
