# Supplementary material for: Participating in extracurricular activities and school sports during the COVID-19 pandemic: Associations with child and youth mental health
Source: Front Sports Act Living. 2022 Aug 29;4:936041. doi: 10.3389/fspor.2022.936041 (PMC9464933; doi:10.3389/fspor.2022.936041)
Supplement: Supplementary file 1 [file Data_Sheet_1.doc]

**Supplemental Table 1**

*Chi-Square Tables for Pre-COVID and During-COVID Participation in Extracurriculars and Sports*

| **ECAs** | **Variable** | **Categories** | **Pre-COVID Participation** | **During-COVID Participation** |
| --- | --- | --- | --- | --- |
|  | Sex at birth | Male | 268 (52%) | 43 (43%) |
|  |  | Female | 244 (48%) | 56 (57%) |
|  |  | **Pearson Chi-Square** | χ^2^ (1, *N* = 877) = 2.83, *p* = .09 | **χ^2^ (1, *N* = 628) = 7.79,**  ***p* = .01*** |
|  | Ethnicity | European/North American | 310 (60%) | 53 (54%) |
|  |  | Non-European/North American | 100 (20%) | 27 (27%) |
|  |  | Multiple | 90 (18%) | 17 (17%) |
|  |  | **Pearson Chi-Square** | χ^2^ (3, *N* = 879) = 6.57, *p* = .09 | χ^2^ (3, *N* = 629) = 7.19,  *p* = .07 |
|  | Household Income | <$80,000 | 113 (26%) | 27 (32%) |
|  |  | >$80,000 | 323 (74%) | 57 (68%) |
|  |  | **Pearson Chi-Square** | χ^2^ (1, *N* = 753) = 3.88, *p* = .05 | χ^2^ (1, *N* = 545) = 1.06,  *p* = .30 |
|  | Pre-COVID MH/NDD diagnosis | MH/NDD Diagnoses | 275 (54%) | 46 (46%) |
|  |  | None | 238 (46%) | 53 (54%) |
|  |  | **Pearson Chi-Square** | χ^2^ (1, *N* = 879) = .31,  *p* = .58 | χ^2^ (1, *N* = 629) = .01,  *p* = .92 |
| **Sports** | Sex at birth | Male | 295 (58%) | 99 (59%) |
|  |  | Female | 215 (42%) | 68 (41%) |
|  |  | **Pearson Chi-Square** | **χ^2^ (1, *N* = 878) = 6.06,**  ***p* = .01*** | χ^2^ (1, *N* = 616) = .73,  *p* = .40 |
|  | Ethnicity | European/North American | 298 (58%) | 85 (51%) |
|  |  | Non-European/North American | 109 (21%) | 44 (26%) |
|  |  | Multiple | 91 (19%) | 33 (20%) |
|  |  | **Pearson Chi-Square** | **χ^2^ (3, *N* = 880) = 17.91**  ***p* <.001*** | **χ^2^ (3, *N* = 617) = 12.62,**  ***p* <.01*** |
|  | Household Income | <$80,000 | 119 (28%) | 45 (31%) |
|  |  | >$80,000 | 314 (72%) | 100 (69%) |
|  |  | **Pearson Chi-Square** | χ^2^ (1, *N* = 753) = .57,  *p* = .45 | χ^2^ (1, *N* = 5.38) = 1.24,  *p* = .27 |
|  | Pre-COVID MH/NDD diagnosis | MH/NDD Diagnoses | 274 (54%) | 79 (47%) |
|  |  | None | 237 (46%) | 88 (53%) |
|  |  | **Pearson Chi-Square** | χ^2^ (1, *N* = 880) = 3.74, *p* = .05 | **χ^2^ (1, *N* = 617) = 10.82,**  ***p* = .001*** |

**Supplemental Table 2**

*Sample Characteristics and Descriptive Statistics by Pre-COVID and During-COVID Extracurricular Activity and Sport Participants and Non-Participants*

| **Pre-COVID Participant and Non-Participant Characteristics** | | | | | |
| --- | --- | --- | --- | --- | --- |
|  | | **Pre-COVID ECA Participants**  **(*n* = 513)** | **Pre-COVID ECA Non-Participants**  **(*n* = 366)** | **Pre-COVID Sport Participants**  **(*n* = 511)** | **Pre-COVID Sport Non-Participants**  **(*n* = 369)** |
| **Covariate** | **Categories** |  |  |  | |
| **Age** | | *M* = 10.76 years  (*SD* = 3.20) | *M* = 10.90 years  (*SD* = 3.47) | *M* = 10.42 years  (*SD* = 3.01) | *M* = 11.34 years  (*SD* = 3.67) |
| **Ethnicity** | European/North American | 310  (60%) | 247  (67%) | 298  (58%) | 260  (70%) |
|  | Non-European/North American | 100  (19%) | 49  (13%) | 109  (21%) | 42  (11%) |
|  | Multiple | 90  (18%) | 60  (17%) | 91  (18%) | 58  (16%) |
|  | Missing | 13  (3%) | 10  (3%) | 13  (3%) | 9  (2%) |
| **Household**  **Income** | <$80,000 | 113  (22%) | 103  (28%) | 119  (23%) | 96  (26%) |
|  | >$80,000 | 323  (63%) | 214  (58%) | 314  (61%) | 224  (61%) |
|  | Missing | 77  (15%) | 49  (13%) | 78  (15%) | 49  (13%) |
| **Pre-COVID Participation**  **(2019-2020)** | Never/once a month | - |  | - |  |
|  | Once a week | 199  (39%) |  | 121  (24%) |  |
|  | A few times a week | 209  (41%) |  | 214  (42%) |  |
|  | Most days | 105  (20%) |  | 176  (34%) |  |
| **During-COVID Participant and Non-Participant Characteristics** | | | | | |
|  | | **During-COVID**  **ECA Participants**  **(*n* = 99)** | **During-COVID**  **ECA Non - Participants**  **(*n* = 530)** | **During-COVID**  **Sport Participants (*n* = 167)** | **During-COVID**  **Sport Non- Participants (*n* = 450)** |
| **Covariate** | **Categories** |  |  |  | |
| **Age** | | *M* = 10.77 years  (*SD* = 3.37) | *M* = 10.92 years  (*SD* = 3.28) | *M* = 9.55  years  (*SD* = 2.40) | *M* = 11.03 years  (*SD* = 3.32) |
| **Ethnicity** | European/North American | 53  (54%) | 339  (64%) | 85  (51%) | 294  (65%) |
|  | Non-European/North American | 27  (28%) | 87  (16%) | 44  (26%) | 70  (16%) |
|  | Multiple | 17  (17%) | 88  (17%) | 33  (20%) | 75  (17%) |
|  | Missing | 1  (1%) | 16  (3%) | 5  (3%) | 11  (2%) |
| **Household**  **Income** | <$80,000 | 27  (28%) | 123  (23%) | 45  (27%) | 103  (23%) |
|  | >$80,000 | 57  (58%) | 338  (64%) | 100  (60%) | 290  (64%) |
|  | Missing | 15  (15%) | 69  (13%) | 22  (13%) | 57  (13%) |
| **Pre-COVID**  **Participation**  **(2019-2020)** | Never/once a month | 7  (7%) | 224  (42%) | 2  (1%) | 204  (45%) |
|  | Once a week | 20  (20%) | 127  (24%) | 19  (11%) | 69  (15%) |
|  | A few times a week | 44  (45%) | 122  (23%) | 73  (44%) | 100  (22%) |
|  | Most days | 27  (27%) | 55  (10%) | 72  (43%) | 74  (16%) |
|  | Missing | 1  (1%) | 2  (-) | 1  (1%) | 3  (1%) |

*Note:* Household income represents annual income in Canadian dollars

**Supplemental Table 3**

*Case Complete Hierarchical Linear Regression Analyses for Extracurriculars and Sports for Blocks 1, 2, and 3*

|  |  | **Depression** | | | | | | **Anxiety** | | | | | | **Hyperactivity** | | | | | | **Inattention** | | | | | |
| --- | --- | --- | --- | --- | --- | --- | --- | --- | --- | --- | --- | --- | --- | --- | --- | --- | --- | --- | --- | --- | --- | --- | --- | --- | --- |
| EA | Variable | ***β*** | **SE** | **LCI,**  **UCI** | ***p*** | ***R^2^*** | **Δ*R^2^*** | ***β*** | **SE** | **LCI,**  **UCI** | ***p*** | ***R^2^*** | **Δ*R^2^*** | ***β*** | **SE** | **LCI,**  **UCI** | ***p*** | ***R^2^*** | **Δ*R^2^*** | ***β*** | **SE** | **LCI,**  **UCI** | ***p*** | ***R^2^*** | **Δ*R^2^*** |
|  | ***Block 1*** | | | | | .01 | *-* |  | | | | .03 | **-** |  | | | | .01 | **-** |  | | | | .01 | - |
|  | Intercept | **-** | **3.42** | **51.51,**  **65.94** | **<.001^*^** |  | | **-** | **.18** | **-.85,**  **-.14** | **.007^*^** |  | | **-** | **1.93** | **.04,**  **7.64** | **<.05^*^** |  | | - | 2.08 | -3.95,  4.24 | .94 |  | |
|  | Age | .09 | .27 | -.10,  1.05 | .05 |  |  | **.18** | **.01** | **.03,**  **.08** | **<.01^*^** |  |  | -.05 | .15 | -.49,  .11 | .21 |  |  | **.10** | **.16** | **.04,**  **.68** | **.03^*^** |  |  |
|  | Income | -.07 | 1.91 | -6.51,  .98 | .15 |  |  | -.07 | .11 | -.37,  .05 | .13 |  |  | **-.09** | **1.13** | **-4.47,**  **-.02** | **<.05^*^** |  |  | -.06 | 1.22 | -4.15,  .64 | .15 |  |  |
|  | Ethnicity | .01 | 1.05 | -1.93,  2.19 | .90 |  |  | -.03 | .06 | -.14,  .08 | .60 |  |  | .02 | .61 | -.97,  1.45 | .70 |  |  | .02 | .66 | -.95,  1.64 | .60 |  |  |
|  | ***Block 2*** | | | | | .01 | .00 |  | | | | .03 | .00 |  | | | | **.03** | **.02^*^** |  | | | | **.04** | **.03^*^** |
|  | Intercept | **-** | **3.62** | **51.70, 65.91** | **<.001^*^** |  | | **-** | **.20** | **-.81,**  **-.04** | **.03^*^** |  | | **-** | **2.04** | **1.77,**  **9.08** | **.005^*^** |  | | - | 2.17 | -.73,  7.80 | .10 |  | |
|  | Age | .09 | .27 | -.02,  1.05 | .06 |  |  | **.18** | **.01** | **.03,**  **.08** | **<.01^*^** |  |  | -.06 | .15 | -.52,  .08 | .14 |  |  | .08 | .16 | -.01,  .63 | .06 |  |  |
|  | Income | -.07 | 1.92 | -6.44,  1.10 | .16 |  |  | -.07 | .11 | -.35,  .06 | .17 |  |  | -.08 | 1.13 | -4.23,  .21 | .08 |  |  | -.05 | 1.20 | -3.69,  1.03 | .27 |  |  |
|  | Ethnicity | .01 | 1.05 | -1.91,  2.23 | .88 |  |  | -.02 | .06 | -.14,  .09 | .65 |  |  | .03 | .61 | -.84,  1.56 | .56 |  |  | .04 | .65 | -.72,  1.83 | .39 |  |  |
|  | Pre-COVID  participation | -.04 | 1.79 | -4.41,  2.63 | .62 |  |  | -.05 | 1.00 | -.30,  .09 | .28 |  |  | **-.12** | **1.04** | **-4.96,**  **.11** | **.005^*^** |  |  | **-.20** | **1.10** | **-7.29,**  **-2.95** | **<.001^*^** |  |  |
|  | ***Block 3*** | | | | | **.04** | **.03^*^** |  | | | | .03 | .00 |  | | | | .03 | .00 |  | | | | **.05** | .01 |
|  | Intercept | **-** | **3.56** | **51.86,**  **65.85** | **<.001^*^** |  | | - | .20 | -.81,  -.04 | .03 |  | | **-** | **2.04** | **1.81,**  **9.83** | **.005^*^** |  | | - | 2.16 | -.66,  7.85 | .10 |  | |
|  | Age | **.10** | **.27** | **.02,**  **1.08** | **.04^*^** |  |  | **.18** | **.01** | **.03,**  **.08** | **<.01^*^** |  |  | -.06 | .15 | -.52,  .08 | .15 |  |  | **.08** | **.16** | **.00,**  **.63** | **<.05*** |  |  |
|  | Income | -.08 | 1.89 | -6.80,  .64 | .10 |  |  | -.07 | .11 | -.36,  .05 | .15 |  |  | -.08 | 1.13 | -4.34,  .11 | .06 |  |  | -.05 | 1.20 | -3.86,  .86 | .21 |  |  |
|  | Ethnicity | .01 | 1.04 | -1.78,  2.29 | .80 |  |  | -.02 | .06 | -.14,  .09 | .69 |  |  | .03 | .61 | -.82,  1.58 | .53 |  |  | .04 | .65 | -.67,  1.87 | .36 |  |  |
|  | Pre-COVID  participation | .03 | 1.84 | -2.56,  4.56 | .57 |  |  | -.04 | .10 | -.27,  .13 | .48 |  |  | **-.10** | **1.09** | **-4.62**  **-.36** | **.02^*^** |  |  | **-.17** | **1.15** | **-6.71,**  **-2.20** | **<.001^*^** |  |  |
|  | During-COVID  participation | **-.19** | **2.40** | **-13.87,**  **-4.42** | **<.001^*^** |  |  | -.06 | .13 | -.42,  .10 | .23 |  |  | -.06 | 1.45 | -4.46  .85 | .17 |  |  | **-.09** | **1.54** | **-6.16,**  **-2.20** | **.04^*^** |  |  |
|  |  | | | | | | | | | | | | | | | | | | | | | | | | |
| Sports | Variable | ***β*** | **SE** | **LCI,**  **UCI** | ***p*** | ***R^2^*** | **Δ*R^2^*** | ***β*** | **SE** | **LCI,**  **UCI** | ***p*** | ***R^2^*** | **Δ*R^2^*** | ***β*** | **SE** | **LCI,**  **UCI** | ***p*** | ***R^2^*** | **Δ*R^2^*** | ***β*** | **SE** | **LCI,**  **UCI** | ***p*** | ***R^2^*** | **Δ*R^2^*** |
|  | ***Block 1*** | | | | | .01 | - |  | | | | .03 | - |  | | | | .01 | **-** |  | | | | .01 | - |
|  | Intercept | **-** | **3.70** | **52.83,**  **67.36** | **<.001*** |  | | **-** | **.19** | **-.78,**  **-.04** | **.03^*^** |  | | **-** | **1.92** | **.86,**  **8.38** | **.02^*^** |  | | - | 2.09 | -3.88,  4.33 | .92 |  | |
|  | Age | .08 | .30 | -.09,  1.10 | .09 |  |  | **.16** | **.02** | **.02,**  **.08** | **<.01^*^** |  |  | -.06 | .15 | -.50,  .11 | .21 |  |  | **.11** | **.17** | **.10,**  **.76** | **.01^*^** |  |  |
|  | Income | -.08 | 2.02 | -7.93,  .56 | .09 |  |  | -.06 | .11 | -.35,  .07 | .18 |  |  | **-.11** | **1.11** | **-4.94,**  **-.57** | **.01^*^** |  |  | -.08 | 1.21 | -4.68,  .08 | .06 |  |  |
|  | Ethnicity | -.01 | 1.13 | -2.51,  1.91 | .79 |  |  | -.06 | .06 | -.18,  .05 | .24 |  |  | .01 | .60 | -1.08,  1.29 | .86 |  |  | .02 | .66 | -.95,  1.63 | .60 |  |  |
|  | ***Block 2*** | | | | | .00 | .00 |  | | | | **.05** | **.02^*^** |  | | | | .01 | .00 |  | | | | **.02** | **.01^*^** |
|  | Intercept | **-** | **4.08** | **53.23,**  **69.27** | **<.001^*^** |  | | - | .21 | -.53,  .29 | .57 |  | | **-** | **2.14** | **.51,**  **8.91** | **.03^*^** |  | | - | 2.32 | -2.20,  **6.91** | .31 |  | |
|  | Age | .08 | .30 | -.13,  1.07 | .12 |  |  | **.13** | **.02** | **.01,**  **.07** | **.007^*^** |  |  | -.06 | .16 | -.51,  .11 | .21 |  |  | **.09** | **.17** | **.03,**  **.70** | **.03^*^** |  |  |
|  | Income | -.08 | 2.03 | -7.34,  .63 | .10 |  |  | -.06 | .11 | -.34,  .08 | .22 |  |  | **-.11** | **1.11** | **-4.94,**  **-.56** | **.01^*^** |  |  | -.08 | 1.21 | -4.56,  .19 | .07 |  |  |
|  | Ethnicity | -.01 | 1.14 | -2.44,  2.02 | .85 |  |  | -.04 | .06 | -.16,  .06 | .39 |  |  | .01 | .61 | -1.08,  1.31 | .85 |  |  | .03 | .66 | -.80,  1.79 | .45 |  |  |
|  | Pre-COVID  participation | -.03 | 1.96 | -5.18,  2.54 | .50 |  |  | **-.15** | **.10** | **-.52,**  **-.16** | **.002^*^** |  |  | .00 | 1.07 | -2.21,  1.99 | .92 |  |  | **-.09** | **1.16** | **-4.69,**  **-.15** | **.04^*^** |  |  |
|  | ***Block 3*** | | | | | **.02** | **.02^*^** |  | | | | .05 | .00 |  | | | | .01 | .00 |  | | | | .02 | .01 |
|  | Intercept | **-** | **4.08** | **54.32,**  **70.37** | **<.001^*^** |  | | - | .21 | -.51,  .32 | .66 |  | | - | 2.16 | .89,  9.37 | .02^*^ |  | | - | 2.34 | -1.65,  7.54 | .21 |  | |
|  | Age | .06 | .31 | -.23,  .98 | .22 |  |  | **.12** | **.02** | **.01,**  **.07** | **.01^*^** |  |  | -.07 | .16 | -.54,  .08 | .14 |  |  | .08 | .17 | -.02,  .65 | .07 |  |  |
|  | Income | -.08 | 2.02 | -7.37,  .56 | .09 |  |  | -.06 | .11 | -.34,  .08 | .22 |  |  | **-.11** | **1.11** | **-5.00,**  **-.62** | **.01^*^** |  |  | -.08 | 1.21 | -4.65,  .10 | .06 |  |  |
|  | Ethnicity | -.01 | 1.13 | -2.35,  2.09 | .91 |  |  | -.04 | .06 | -.16,  .07 | .41 |  |  | .01 | .61 | -1.03,  1.36 | .79 |  |  | .04 | .66 | -.73,  1.86 | .40 |  |  |
|  | Pre-COVID  participation | .02 | 2.13 | -3.46,  -4.90 | .74 |  |  | **-.13** | **.11** | **-.50,**  **-.06** | **.01^*^** |  |  | .02 | 1.16 | -1.77,  2.78 | .66 |  |  | **-.06** | **1.25** | **-4.03,**  **.89** | **.21** |  |  |
|  | During-COVID  participation | **-.13** | **2.23** | **-9.77,**  **-.99** | **.02^*^** |  |  | -.05 | .12 | -.34,  .12 | .34 |  |  | -.07 | 1.24 | -4.13,  .74 | .17 |  |  | -.08 | 1.34 | -5.00,  .28 | .08 |  |  |
|  |  | | | | | | | | | | | | | | | | | | | | | | | | |

*Note:* **Block 2** controlled for age, income, ethnicity, with pre-COVID participation as the independent variable; **Block 3** controlled for age, income, ethnicity, pre-COVID participation with during-COVID participation as the independent variable; Age = child age in years; Income = annual household income in Canadian dollars; Depression was measured by the Revised Child and Anxiety Depression Scale – Parent Version (RCADS-P); Anxiety was measured by the Screen for Child Anxiety Related Disorders (SCARED); Hyperactivity and inattention were measured by subscales of the Strengths and Weaknesses of Attention-Deficit/Hyperactivity Disorder Symptoms and Normal Behaviour Scale (
